
JOURNAL INFORMATION
==============================
NLM Title Abbreviation: ScientificWorldJournal
Journal ID: ScientificWorldJournal
NLM Journal ID: 101131163
Journal ID: TSWJ

The Scientific World Journal

ISSN: 2356-6140
EISSN: 1537-744X
Publisher: Wiley

ARTICLE INFORMATION
==============================
PMCID: PMC5849069
PMID: 19112542
DOI: 10.1100/tsw.2008.164
Article ID: 563254
Article version: 1
Subjects: Directions in Science

Inhibition of Steroid Sulfatase Activity in Endometriotic Implants by STX64 (667Coumate): A Potential New Therapy

Fusi L. 1
Purohit A. 2
Brosens J. 1
Woo L. W. L. 3
Potter B. V. L. 3
Reed M. J. 2 *
1 Institute of Reproductive and Developmental Biology, Imperial College London, Hammersmith Hospital, Du Cane Road, London, W12 0NN, UK
2 Endocrinology and Metabolic Medicine and Sterix Ltd., Imperial College London, St. Mary's Hospital, Praed Street, London,W2 1NY, UK
3 Medicinal Chemistry, Department of Pharmacy and Pharmacology and Sterix Ltd., University of Bath, Claverton Down, Bath, BA2 7AY, UK
*M. J. Reed: b.v.l.potter@bath.ac.ukAcademic Editor: Martin Goette

Publication date: 2008
Electronic publication date: 2008 Dec 25
Volume: 8
First page: 1325
Last page: 1327
Received 2008 Aug 20; Revised 2008 Dec 10; Accepted 2008 Dec 17
Copyright: Copyright © 2008 L. Fusi et al.
Copyright year: 2008
License: This is an open access article distributed under the Creative Commons Attribution License, which permits unrestricted use, distribution, and reproduction in any medium, provided the original work is properly cited.
License URL: https://creativecommons.org/licenses/by/3.0/

Keywords: endometriosis, steroid sulfatase, steroid sulfatase inhibitors, STX64, 667Coumate

Funding: Sterix Ltd.

==============================
Directions in Science
*TheScientificWorldJOURNAL* (2008) 8, 1325–1327
ISSN 1537-744X; DOI 10.1100/tsw.2008.164

TheScientificWorldJOURNAL
www.thescientificworld.com

### Inhibition of Steroid Sulfatase Activity in Endometriotic Implants by STX64 (667Coumate): A Potential New Therapy

L. Fusi[1], A. Purohit[2], J. Brosens[1], L.W.L. Woo[3], B.V.L. Potter[3], and M.J. Reed[2,]*

*[1]Institute of Reproductive and Developmental Biology, Imperial College London, Hammersmith Hospital, Du Cane Road, London, W12 0NN, U.K.; [2]Endocrinology and Metabolic Medicine and Sterix Ltd., Imperial College London, St. Mary's Hospital, Praed Street, London, W2 1NY, U.K.; [3]Medicinal Chemistry, Department of Pharmacy and Pharmacology and Sterix Ltd., University of Bath, Claverton Down, Bath, BA2 7AY, U.K.*

**E-mail**: l.fusi@imperial.ac.uk; a.purohit@imperial.ac.uk; j.brosens@imperial.ac.uk; m.reed@imperial.ac.uk; l.w.l.woo@bath.ac.uk; b.v.l.potter@bath.ac.uk

*Received August 20, 2008; Revised December 10, 2008; Accepted December 17, 2008; Published December 25, 2008*

**KEYWORDS**: endometriosis, steroid sulfatase, steroid sulfatase inhibitors, STX64, 667Coumate

Endometriosis is an increasingly common gynecological disorder characterized by the growth of ectopic endometrial tissue outside the endometrial cavity. The majority of the lesions are localized within the peritoneal cavity and, in particular, on the peritoneum, the ovaries, the pouch of Douglas, and the bowels. The disease affects a large number of women throughout the world, and it has been estimated that between 5 and 10% of women of reproductive age have endometriosis[1], although this is likely to be an underestimate of the true prevalence, which is unknown.

The etiology of endometriosis is not well understood, but retrograde menstruation, as first proposed by Sampson in 1927[2], appears to be the most accepted theory. In addition to this, other factors, such as altered immune response, metaplastic transformation, and vascular spread, have been suggested to explain the different aspects of endometriosis[3,4].

The main symptoms of the disease are severe pelvic pain, dyspareunia, dysmenorrhea, and menstrual disturbances. The disease is also a frequent finding in women complaining of infertility, although the precise relationship between the two conditions remains to be defined. The gold standard for diagnosis is laparoscopic visualization of the pelvis, usually carried out for severe symptoms or for infertility workup.

A recent study by our group published in *Human Reproduction*[5] has shown that high levels of steroid sulfatase (STS) activity were present in both eutopic and ectopic endometrium in women with endometriosis. The study also showed that the addition of a new STS inhibitor, STX64 (BN83495, 667Coumate), to the endometrial tissue almost completely suppressed enzyme activity, indicating a possible therapeutic potential for this compound in endometriotic lesions.

Estrogens are thought to play an important role in the growth and development of endometriosis[3], and current medical treatments are based on ovarian estrogen suppression or modulation. Treatments include estrogen-progestin preparations, progestins, GnRH analogues, and androgen-derived drugs like

*Corresponding author.
©2008 with author.
Published by TheScientificWorld; www.thescientificworld.com

Danazol. The effect of these therapies is simply to control the symptoms, but their relief is limited and associated with side effects.

Recent data have suggested that, in addition to ovarian estrogens, local production of estrogens by the endometrial ectopic tissue could affect disease growth and progression. This is thought to involve the aromatase pathway. Transcripts for aromatase P450 encoding the enzyme that converts androgens to estrone (CYP19A1) have been isolated in samples of endometriotic tissue[6], and high levels of aromatase activity have been detected in cells cultured from ectopic endometrial tissue[7]. This discovery has led to the development and use of aromatase inhibitors for the treatment of the disease[8,9].

STS is another enzyme involved in intracrine production of estrogens, through conversion of the estrone and estradiol sulfates to their unconjugated forms[10]. STS mRNA expression was found to be five times higher in ovarian endometriosis than in normal endometrium[11], and the expression of the estrogen-inactivating enzyme, steroid sulfotransferase, was found to be absent in some endometriotic tissue samples. A further mechanism potentiating the local effect of estrogens is related to the lack of expression of another estradiol-metabolizing enzyme, 17β-hydroxysteroid-dehydrogenase Type 2, in endometriotic tissue[12]. This enzyme oxidizes biologically active estradiol to the less potent estrogen, estrone, which has a reduced affinity for the estrogen receptor. The activities of enzymes involved in local estrogen production are stimulated by cytokines, such as TNFα and IL-6[13,14], which are abundantly present in the peritoneal fluid of women with endometriosis. It appears, therefore, that the STS pathway is another enzymatic mechanism to ensure, via a positive feedback loop, both production and maintenance of a high level of local estrogens to help the development of endometriosis.

Several irreversible STS inhibitors, such as STX64 and estrone-3-*O*-sulfamate, have been synthesized and found to be active *in vivo*[15,16]. One of these compounds, STX64, has been used to treat metastatic breast cancer in postmenopausal women and was found to suppress STS activity in breast tumors and peripheral tissue almost completely, and to reduce serum estrone and estradiol concentrations significantly[17]. The effects of these new powerful compounds suggest that they could be used to treat endometriosis.

Our study[5] was designed to measure and compare aromatase and STS activities in eutopic and ectopic endometrium in women with endometriosis, to correlate this with severity of disease, and to assess the effect of STX64 on STS activity in endometrial tissue. Paired samples of eutopic and ectopic endometrium were collected, snap frozen, and subsequently analyzed. The aromatase and STS activities were measured, with STS activity being measured in the absence or presence of the STS inhibitor (STX64). Endometriosis was clinically classified according to the revised American Fertility Society classification[18]. We found very significant levels of STS activity in endometriotic implants. Although both aromatase and STS activities were present in all samples, STS activity was significantly higher and less variable than aromatase, and the activity was also directly correlated with severity of disease. Importantly, we found that STX64 almost completely abrogated STS activity in all the samples (>99%).

In summary, endometriosis is a common and debilitating condition of women in their reproductive age, and for which present medical treatments provide only limited control of symptoms. The realization that local production of estrogens could be important for the development of the disease has introduced the need for more targeted treatments. The finding that local enzymes, such as aromatase and STS, are present in endometriotic tissue has heralded a new era for the development of such treatments. New powerful STS inhibitors, like STX64, are presently being evaluated for the control of estrogen-dependent diseases, such as breast cancer, and preliminary studies are demonstrating their effectiveness in suppressing enzyme activity and local estrogen production. Our study indicates that they could also be used in the treatment of endometriosis. Further studies are needed to assess their effectiveness *in vivo* more precisely and, at the same time, their safety profile and range of potential side effects.

### ACKNOWLEDGMENTS

This work was supported by Sterix Ltd., a member of the Ipsen Group.

### REFERENCES

1. Vigano, P., Parazzini, F., Somigliana, E., and Vercellini, P. (2004) Endometriosis: epidemiology and aetiological factors. *Best Practice Res. Clin. Obstet. Gynaecol.* **18**, 177–200.
2. Sampson, J.A. (1927) Peritoneal endometriosis due to menstrual dissemination of endometrial tissue into the peritoneal cavity. *Am. J. Obstet. Gynecol*. **14**, 422–425.
3. Nap, A., Groothuis, P.G., Demir, A.Y., Evers, J.L.H., and Dunselman, G.A.J. (2004) Pathogenesis of endometriosis. *Best Practice Res. Clin. Obstet. Gynaecol.* **18**, 233–244.
4. Halme, I., White, C., Kauma, S., Estes, J., and Haskill, S. (1988) Peritoneal macrophages from patients with endometriosis release growth factor activity *in vitro*. *J. Clin. Endocrinol. Metab.* **66**, 1044–1049.
5. Purohit, A., Fusi, L., Brosens, J., Woo, L.W.L., Potter, B.V.L., and Reed, M.J. (2008) Inhibition of steroid sulphatase activity in endometriotic implants by 667 COUMATE: a potential new therapy. *Hum. Reprod.* **23**, 290–297.
6. Noble, L.S., Simpson, E.R., Johns, A., and Bulun, S.E. (1996) Aromatase expression in endometriosis. *J. Clin. Endocrinol. Metab*. **81**, 174–179.
7. Noble, L.S., Takayama, K., Zeitoun, K.M., Putman, J.M., Johns, D.A., Hinselwood, M.M., Agarwal, V.R., Zhao, Y., Carr, B.R., and Bulun, S.E. (1997) Prostaglandin E2 stimulates aromatase expression in endometriosis derived stromal cells. *J. Clin. Endocrinol. Metab.* **82,** 600–602.
8. Bulun, S.E., Zeitoun, K.M., Takayama, K., Simpson, E.R., and Sasano, H. (2000) Aromatase as a therapeutic agent in endometriosis. *Trends Endocrinol. Metab.* **11**, 22–27.
9. Takayama, K., Zeitoun, K., Gunby, R.T., Sasano, H., Carr, B.R., and Bulun, S.E. (1998) Treatment of severe postmenopausal endometriosis with an aromatase inhibitor. *Fertil. Steril.* **69**, 709–713.
10. Reed, M.J., Purohit, A., Woo, L.W.L., Newman, S.P., and Potter, B.V.L. (2005) Steroid sulphatase: molecular biology, regulation, and inhibition. *Endocr. Rev.* **26**, 171–202.
11. Smuc, T., Pucely, M.R., Sinkovec, J., Husen, B., Thole, H., and Rizner, T.L. (2007) Expression analysis of the genes involved in estradiol and progesterone action in human ovarian endometriosis. *Gynecol. Endocrinol.* **23**, 105–111.
12. Zeitoun, K., Takayama, K., Sasano, H., Suzuki, T., Moghrabi, N., Anderson, S., Johns, A., Meng, L., Putman, M., Carr, B., et al. (1998) Deficient 17β-hydroxysteroid dehydrogenase Type 2 expression in endometriosis: failure to metabolize 17β estradiol. *J. Clin. Endocrinol. Metab.* **83**, 4474–4480.
13. Purohit, A., Wang, D.Y., Ghilchik, M.W., and Reed, M.J. (1996) Regulation of aromatase and sulphatase in breast cancer cells. *J. Endocrinol*. **150(Suppl.),** S56–S71.
14. Reed, M.J. and Purohit, A. (1997) Breast cancer and the role of cytokines in regulating estrogen synthesis: an emerging hypothesis. *Endocr. Rev*. **18**, 701–715.
15. Woo, L.W.L., Purohit, A., Reed, M.J., and Potter, B.V.L. (1996) Active site-directed inhibition of estrone sulphatase by non-steroidal coumarin sulphamates. *J. Med. Chem.* **39**, 1349–1351.
16. Woo, L.W.L., Purohit, A., Malini, B., Reed, M.J., and Potter, B.V.L. (2000) Potent active site-directed inhibitors of steroid sulphatase by tryciclic coumarin-base sulphamates. *Chem. Biol.* **7,** 773–791.
17. Stanway, S.J., Purohit, A., Woo, L.W.L., et al. (2006) Phase I study of STX64 (667 COUMATE) in breast cancer patients: the first study of a steroid sulphatase inhibitor. *Clin. Cancer Res*. **12**, 1585–1592.
18. (1985) Revised American Fertility Society classification for endometriosis. *Fertil. Steril.* **43**, 351–352.

**This article should be cited as follows:**

Fusi, L., Purohit, A., Brosens, J., Woo, L.W.L., Potter, B.V.L., and Reed, M.J. (2008) Inhibition of steroid sulfatase activity in endometriotic implants by STX64 (667Coumate): a potential new therapy. *TheScientificWorldJOURNAL* **8**, 1325–1327. DOI 10.1100/tsw.2008.164.
